# Mechanoautophagy: Synergies Between Autophagy and Cell Mechanotransduction at Adhesive Complexes

**DOI:** 10.3389/fcell.2022.917662

**Published:** 2022-06-01

**Authors:** Andrea Ravasio, Eugenia Morselli, Cristina Bertocchi

**Affiliations:** ^1^ Institute for Biological and Medical Engineering Schools of Engineering, Medicine and Biological Sciences, Pontificia Universidad Católica de Chile, Santiago, Chile; ^2^ Department of Basic Sciences, Faculty of Medicine and Sciences, Universidad San Sebastián, Santiago, Chile; ^3^ Laboratory for Molecular Mechanics of Cell Adhesion, Department of Physiology Pontificia Universidad Católica de Chile, Santiago, Chile

**Keywords:** mechanoautophagy, Extracellular Matrix, focal adhesion, cadherin mediated adhesion, Autophagy

## Abstract

Cells are exposed and respond to various mechanical forces and physical cues stemming from their environment. This interaction has been seen to differentially regulate various cellular processes for maintenance of homeostasis, of which autophagy represents one of the major players. In addition, autophagy has been suggested to regulate mechanical functions of the cells including their interaction with the environment. In this minireview, we summarize the state of the art of the fascinating interplay between autophagy and the mechanotransduction machinery associated with cell adhesions, that we name ¨Mechanoautophagy¨

## 1 Introduction

How the mechanics of cellular environment influence biological properties is an emerging but yet poorly understood field of investigation. This is particularly true for macroautophagy (herein referred to as autophagy), a dynamic clearance process whereby cellular components, such as misfolded proteins, abnormal protein aggregates and damaged organelles, are sequestered and digested by lysosomes for degradation and recycling (Boya et al., 2013; Ortiz-Rodriguez and Arevalo, 2020; Aman et al., 2021; Hernández-Cáceres et al., 2021). At the molecular level, activation of the autophagic pathway begins with the dissociation of the ULK1/mTORC1 complex, where Unc-51 Like Autophagy Activating Kinase (ULK)1 initiates the recruitment of the autophagic machinery when freed from the inhibitory effect of the kinase mammalian target of rapamycin (mTORC)1 (Shang and Wang, 2011; Park et al., 2016). Downstream of inactivation of mTORC1-repressor function, there are around 20 autophagy-related proteins (collectively called ATGs) that initiate the process by recruiting the necessary machinery for phagophore formation (Geng et al., 2008), (Gómez-Sánchez et al., 2021). Following these initial steps, the phagophore elongates and closes into a double-membrane organelle, called autophagosome that matures into an autolysosome through fusion with lysosomes (Lőrincz and Juhász, 2020). This last step enables digestion of faulty cellular components, recycling of metabolic materials and rejuvenation of the cytosol. A mechanistic description of the whole process can be found in Hernandez et al., Frontiers 2021 (Hernández-Cáceres et al., 2021). In addition to be constantly needed as housekeeping process to maintain cellular homeostasis (Rabinowitz and White, 2010; Galluzzi et al., 2014; Kaur and Debnath, 2015), autophagy is essential during stress response, such as starvation, where, by degrading cytosolic material, autophagy provides nutrients and metabolites necessary for the cell to cope with stress and ensure its survival (Rabinowitz and White, 2010; Das et al., 2012; Müller et al., 2015). Similar stress responses activating autophagy include oxidative stresses, DNA damage and pathogen infection (Filomeni et al., 2015; Eliopoulos et al., 2016; Ravanan et al., 2017; Evans et al., 2018). In addition to these biochemical stresses, we have recently proposed a possible mechanism for autophagy activation in response to mechanical stimuli through involvement of the mechanically activated mTORC2 and its well-known inhibitory effect over mTORC1 (Hernández-Cáceres et al., 2021; Ballesteros‐Álvarez and Andersen, 2021).

Cells are exposed and respond to various mechanical forces and physical cues stemming from their micro- and macro-environment. These include properties of the extracellular matrix (e.g., composition, density, and stiffness), nano and micro-scale geometrical cues (e.g., topography, size, confinement, curvature) that can influence cortex and membrane tension, interaction with neighboring cells (e.g., cell crowding and migratory forces), and the large-scale tissue and organ dynamics (e.g., shear stress, fluid pressure, stretching and compression) (Ingber, 2008; Geiger and Bershadsky, 2002; Iskratsch et al., 2014; Sheetz and Yu, 2018). Mechanical forces are sensed by cells through various mechanosensors, such as adhesion complexes (e.g., adherens junction and focal adhesion), proteins sensing tension and curvature of the plasma membrane (e.g., BAR proteins) and of the cytoskeleton (e.g., filamin), and stretch activated ion channels (e.g., TRP and piezo) (Hernández-Cáceres et al., 2021; Sheetz and Yu, 2018). The mechanical input acting on these mechanosensors triggers cellular responses that may involve direct mechanical responses (largely through cytoskeletal and membrane dynamics) and signaling cascades that convert the mechanical stimulus into a biochemical response (i.e., mechanotransduction) leading to cytoskeletal reorganization, membrane and organelles trafficking, gene expression regulation and consequent modulation of various cellular functions (Iskratsch et al., 2014). Specifically, the interaction between cells and their physical environment regulates positively and negatively the autophagic process (Hernández-Cáceres et al., 2021; King et al., 2011; Dupont and Codogno, 2016; Claude-Taupin et al., 2021; Zhang et al., 2022). On the other hand, autophagic catabolism affects mechanical functions of the cells including their interaction with the environment (Hernández-Cáceres et al., 2021; King et al., 2011; Dupont and Codogno, 2016; Claude-Taupin et al., 2021). In this minireview, we aim to summarize the state of the art of the fascinating interplay between autophagy and the mechanotransduction machinery associated with adhesions that we named ¨Mechanoautophagy¨.

## 2 Extracellular Matrix and Autophagy

The extracellular matrix (ECM) is a dynamic network with different macromolecular composition, structural architecture, and rheological properties that, through its constant remodeling by the cells, contributes to regulating tissue homeostasis (Vogel, 2018). It is indeed this continuous transformation of the ECM that eventually influences a wide array of biological functions (i.e., adhesion and cohesion, proliferation, differentiation, migration, etc) and cellular phenotypes, thus having a dramatic effect on intracellular signaling. This fundamental physiological role implies that the deregulation of such extracellular microniche could lead to diseases. Typically, aberrant ECM organization is observed in various pathological scenarios such as fibrosis and cancer, where ECM composition and rheological properties are altered as compared to the corresponding physiological tissue (Vogel, 2018; Lu et al., 2011).

Specific components associated with the ECM have been reported to play opposing roles on autophagy (Lock and Debnath, 2008; Neill et al., 2014; Schaefer and Dikic, 2021). Inhibitors of autophagy comprise laminin *α*2, an ECM-associated protein, and two proteoglycans, lumican and perlecan. Laminin *α*2 is the heavy chain of the laminin glycoprotein complex and it works as a connector between ECM and the cell membrane in skeletal muscles, Schwann cells, pericytes and astrocytes (Yurchenco et al., 2018). Laminin *α*2 functions as an autophagy inhibitor, as indicated by the increase in autophagic flux in laminin *α*2-deficient muscle cells and by recovery of the typical muscle morphology upon chemical inhibition of laminin *α*2 in congenital muscular dystrophy models (Carmignac et al., 2011; Durbeej, 2015). Lumican has been reported to inhibit autophagy in pancreatic ductal adenocarcinoma through downregulation of AMP-activated protein kinase (AMPK) (Li et al., 2016). Finally, perlecan, as a whole molecule, has been seen to hinder autophagy through mTORC1 activation (Ning et al., 2015). On the other hand, several ECM-associated proteins such as collagen VI, kringle 5, endostatin, and various proteoglycans like decorin, endorepellin, biglycan function as activators of the autophagic process (Nguyen et al., 2007; Nguyen et al., 2009; Gubbiotti and Iozzo, 2015; Castagnaro et al., 2018). Collagen VI, similarly to the associated leucine-rich proteoglycan decorin, has pro-survival and autophagy instructive properties through inactivation of the Akt/mTOR/p70S6K pathway (Castagnaro et al., 2018), and through AMPK *via* the hepatocyte and the epithelial growth factors (HGF/Met and EGF, respectively). Together with decorin, another leucine-rich proteoglycan, biglycan, has been reported to evoke autophagy in macrophages *via* a novel CD44/Toll-like receptor 4 signaling cascade (Poluzzi et al., 2019). Interestingly, while the whole perlecan molecule has inhibitory functions, its c-terminus (AKA endorepellin) enhances autophagy through transcriptional upregulation of pro-autophagic genes such as PEG3, BECN1, and MAP1LC3A (Poluzzi et al., 2014). Kringle 5, the fifth kringle domain in human plasminogen, activates autophagy in a similar manner as endostatin, by enhancing BECN1 expression through β-catenin and Wnt-mediated signaling pathways (Nguyen et al., 2007).

While there are some evidences on how ECM can influence autophagy, little is known about the role of autophagy in regulating cell-ECM interactions. Interestingly, the term ¨secretory autophagy¨ has been coined to indicate the non-lytic autophagic pathway where autophagosomes, instead of fusing with a lysosome, fuse with the plasma membrane and help excrete particulate substrates (Kimura et al., 2017; Ponpuak et al., 2015). The secretion of matrix components could possibly rely on such a mechanism, since deletion of ATG7 in mouse embryonic fibroblast cells produces a deficiency in the expression of collagen I, fibronectin, and periostin (Zhuo et al., 2013). Furthermore, the autophagic process can also influence conventional secretory pathways (i.e., constitutive and regulated secretion) by promoting the translocation of integral membrane proteins to the plasma membrane.

## 3 Focal Adhesions and Autophagy

Mechanical and chemical signals from the extracellular matrix in normal and in pathological conditions are sensed by the integrin-mediated adhesions, also known as focal adhesions (Kanchanawong et al., 2010; Iskratsch et al., 2014). This supramolecular complex physically connects the ECM to the actin cytoskeleton through an intricate plaque of proteins (AKA adhesome network) organized in three distinct layers: a signaling layer composed of transmembrane integrins and adaptor proteins (e.g., paxillin), an intermediate force-transduction layer with mechanotransduction molecules (i.e., talin, vinculin) and signaling molecules (e.g., FAK, Src, PI3K), and, finally, an actin-regulatory layer with actin and actin linker proteins (e.g., filamin, α-actinin) (Kanchanawong et al., 2010; Xia et al., 2019). Mature focal adhesions are highly integrated with the cytoskeleton, as suggested by their presence at the anchor points of actin stress fibers. As such, they are instrumental in transmitting forces internally generated by the actomyosin network to the ECM, and vice versa (Burridge and Guilluy, 2016). Furthermore, focal adhesion assembly normally occurs in actin-rich regions, where clusters of integrins are delivered together by actin polymerization driven by actin retrograde flow (Oakes and Gardel, 2014). Interaction between integrin heterodimers (*α* and *β*) and ECM proteins initiates tension-induced conformational change in integrin cytoplasmic tails with consequent activation of the dimer and its engagement with talin and paxillin (Shattil et al., 2010). The increased tension prompts recruitment of proteins of the signaling layer (e.g., FAK, Src etc.) that, in turn, start the signaling cascade leading to actin polymerization and to the strengthening and growth of the adhesion (Iskratsch et al., 2014). Such a process of conversion of the extracellular mechanical stimuli into biochemical signals (mechanotransduction) is strongly related to several stress responses, including autophagy. One of the first pieces of evidence reporting the bidirectional connection between focal adhesion and autophagy comes from studies on hepatocytes’ osmosensing (Dahl et al., 2003). In this model, integrins have been shown to mediate the activation of Src kinase when anchorage to the extracellular matrix and polarity of hepatocyte was preserved. This interaction triggers the activation of p38MAPK and Erk-1/Erk-2, promoting autophagy and proteolysis (Dahl et al., 2003). This study, was the first that suggested a relation between integrins signaling and autophagic proteolysis, which was then corroborated by more recent studies thatidentified additionaldownstream effectors of the integrin-dependent control of autophagy (Vlahakis and Debnath, 2017). In particular, simply providing detached cells with a laminin-rich ECM, re-establishing cell-ECM contact, abolishes autophagy; this effect is reversed when integrin *β*1 are blocked by using a specific antibody thus inhibiting the FAK and ILK (Integrin Linked Kinase) signaling cascade. Decrease of mechanical forces at the FA, due to detachment from ECM or changes in substrate rheology, leads to dissociation of FAK from integrin and FA (Martino et al., 2018). Soluble FAK can phosphorylate and activate mTORC2 and consequently initiate autophagy (Case et al., 2011). Furthermore, following cell detachment, integrin *β*1 and receptor tyrosine kinase c-Met are removed from the cell membrane and recruited to LC3 autophagic membranes (Barrow-McGee et al., 2016). The pool of internalized integrin *β*1 prompts the c-Met dependent phosphorylation and consecutive activation of ERK1/2, that allows for resistance to anoikis (a programmed cell death occurring upon cell detachment from the ECM (Barrow-McGee et al., 2016)). The activation of autophagy mediated by integrin detachment from ECM, could be thought as a failsafe mechanism to delay the onset of apoptosis and allow cell adaptation and survival (Lock and Debnath, 2008), (Vlahakis and Debnath, 2017), (Anlaş and Nelson, 2020). Unfortunately, this autophagy-mediated survival mechanism could also aid cancer onset and tumor progression (Buchheit et al., 2014).

On the other hand, it has been demonstrated that autophagy plays a crucial role in regulating focal adhesion dynamics. During cell migration, FAs undergo continuous assembling and disassembling cycles that depend on tension and phosphorylation, which is partially mediated by autophagy. For instance, autophagy targets integrin *β*1 during nutrient starvation (Vlahakis and Debnath, 2017) regulating FA dynamics and promoting their turnover (Sharifi et al., 2016). This occurs *via* different pathways involving LC3 and autophagy receptors that target specific FA components such as the selective autophagy cargo adaptor NBR1 that can bind to a variety of FA proteins (i.e., vinculin, FAK, paxillin, and zyxin), and recruits LC3-containing autophagosomes to FAs (Chang et al., 2017), (Kenific and Debnath, 2016). In addition, once phosphorylated by Src, paxillin is also targeted by LC3-containing-autophagosomes *via* its direct association with LC3. Finally, active Src can be targeted by the cargo adaptor, Cbl, which recruits autophagosomes to FA for Src degradation (Chang et al., 2017; Cecconi, 2012). An interesting venue of interplay between FAs and autophagy could also involve regulation of actin contractility and cytoskeletal dynamics. Autophagy can alter these processes by specifically degrading RhoA *via* the autophagic receptor p62/SQSTM1 (Bjørkøy et al., 2009).

## 4 Cell-Cell Adhesions and Autophagy

Besides the adhesion between cells and ECM, integrity, homeostasis and dynamics of cells and tissues are regulated by physical interaction between neighboring cells. Despite the variety of adhesion complexes mediating adhesion and communication between neighboring cells, in this minireview we will focus on adherens junctions (AJs) because of their role as mechanotransducers (Angulo-Urarte et al., 2020). AJs, also known as cadherin-mediated adhesion, mediate force transduction between cells by specialized transmembrane receptors (i.e., cadherins) that are connected to the cytoskeleton *via* a protein complex termed the cadhesome network (Zaidel-Bar, 2013). Superresolution microscopy experiments demonstrated that AJs share remarkable similarities with FAs, including a multilayered architecture (Kanchanawong et al., 2010; Bertocchi et al., 2017) with a signaling layer composed by cadherin, β-catenin, α-catenin, p120-catenin, a force transduction layer where vinculin, zyxin and VASP are and an actin regulatory layer with actin, α-actinin, and eplins (Bertocchi et al., 2017). Due to these similarities, one could expect that a comparable bidirectional control between autophagy and adhesions could be found. However, this is only partially true, and substantial investigations unveiling these interactions are still missing. It has been observed that autophagy-dependent survival was promoted in vascular smooth muscle cells following T-cadherin upregulation and activation of MEK1/2-Erk1/2 (Kyriakakis et al., 2017). Furthermore, it has been demonstrated that force application to E-cadherin adhesion, prompts autophagy through activation of Liver Kinase B1 (LKB1), which recruits AMPK at the site of the AJs (Bays et al., 2017). Similarly to what is observed for FAs, autophagy machinery also contributes to AJs turnover. In particular, it has been observed that in breast cancer, E-cadherin physically interacts with p62/SQSTM1 to mediate LC3 targeting and consequent delivery to LC3-containing autophagosomes (Damiano et al., 2020; Santarosa and Maestro, 2021). Additionally, LC3 has been found to directly interact with β‐catenin to target its degradation (Petherick et al., 2013). Autophagy has been reported to degrade transcription factors SNAIL and SLUG, which control E-to N-cadherin switch during Epithelial to Mesenchymal Transition (EMT) process, *via* binding to the autophagy adaptor p62/SQSTM1 (Bertrand et al., 2015). This results in reduced migration and invasion of cancer cells (i.e. glioblastoma) and leads to reversing EMT. This EMT modulatory role of autophagy has been corroborated by the observation that its deficiency or suppression enhance cell migration, invasion, and proliferation, potentially due to the stabilization of transcription factor Twist1 by p62/SQSTM1 (Qiang and He, 2014). However additional sets of evidence support the opposing view that inhibition of autophagy (either chemically, or by silencing of core autophagy genes such as Beclin1 or ATG7) could foster the expression of epithelial markers, whereas its induction could lead to activation of SNAIL transcription factor and consequently EMT (Chen et al., 2019). Thus, lack of sufficient body of evidence leaves open to debate the effective role and actual importance of autophagy in maintaining tissue homeostasis and in regulating EMT.

## 5 Yes-Associated Protein/Transcriptional Co-Activator with PDZ-Binding Motif Mechanical Response and Autophagy

Yes-associated protein (YAP) and the transcriptional co-activator with PDZ-binding Motif (TAZ) are proto-oncogenes that can modulate gene expression in response to changes of the mechanical environment (Sudol, 1994; Low et al., 2014). Piccolo and co-workers have been the pioners in the study of YAP/TAZ mechanosensing mechanisms and demonstrated changes in localization of these two transcriptional activators, depending on mechanical forces. In particular, they have shown a differential translocation in and out of the nucleus (and consequent activation or inactivation), depending on extracellular matrix stiffness, cell density and cell geometry (Dupont et al., 2011). E-cadherin/catenin complex and integrins function as an upstream regulators of the Hippo signaling pathway in signal transduction in mammalian cells (Kim et al., 2011; Kim and Gumbiner, 2015; Short, 2015; Block et al., 2020); it has been shown that in subconfluent epithelial cell cultures or when cells are seeded on a stiff ECM, YAP and TAZ remain in the nucleus where they promote cell proliferation, through their interaction with TEAD family of transcription factors (Aragona et al., 2013; Panciera et al., 2017). In high-cell density population, where contact between cells is preserved, or when cells are on a soft substrate, YAP and TAZ are not active and localize in the cytoplasm (Zhao et al., 2007). This is part of a self-defending mechanism that allows noncancerous cells to stop proliferating when they contact one another. Such contact inhibition appears deregulated in cancer cells, that bypass the command from cell adhesions and keep on proliferating (Pavel et al., 2018). In different cell types, it has been shown a contrasting effect on YAP/TAZ signaling in response to alteration of autophagy; some studies correlate defect of autophagy with inhibition of YAP/TAZ, failure in modulation of myosin-II gene expression and consecuent loss of F-actin stress fibers. In a feedback loop this loss of F-actin fibers leads to impairment in autophagosome formation by altering the amount of ATG16L1 puncta and by reduced co-localization with ATG9A-LC3 (Pavel et al., 2018). Low cell density induced YAP/TAZ activation in the nucleus, results in actin stress fibers formation and autophagosomes assembly. These results suggest a feedback loop between autophagy and Hyppo pathway.

On the other hand, other groups reported that autophagy alteration, by Beclin silencing or by cloroquine treatment, can induce expression of YAP in cancer cell lines (from lung, breast and colon) (Wang et al., 2019). These opposite responses in different cell types seems to be related to α-catenins levels (Pavel et al., 2021). Interestingly, α-catenins are common key signalling effectors between autophagy and Hyppo pathway; they are known to interact with LC3 and they can inhibit YAP/TAZ signaling. When α-catenins levels are low [i.e., in cancer cells from lung, breast, colon (Sun et al., 2014)], YAP/TAZ activity is increased upon autophagy inhibition, while, YAP/TAZ activity is reduced by autophagy when α-catenin levels are high (Pavel et al., 2021)**.** Viceversa, inhibition of the Hyppo pathway, in response to the physical properties of the cell microenvironment (high cell density) (Pavel et al., 2018) reduces the efficiency of the autophagic flux (Totaro et al., 2019). At last, mTORC1 regulates YAP by mediating its autophagic degradation (Liang et al., 2014), further linking cellular nutrient status to YAP activity (Pocaterra et al., 2020), and strenghtening the hypothesis for crosstalk between the transcriptional coactivatorsYAP/TAZ and autophagy.

**FIGURE1:**
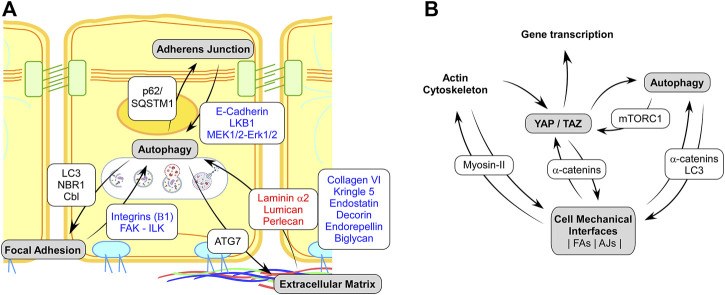
**(A)** Schematic representation of the known interplays between the mechanosensitive cell adhesions complexes and autophagy. Red and blue texts on white background indicate protein showing a negative or positive regulation of autophagy, respectively. Text in black on white background indicates proteins of the autophagy machinery targeting specific disassembly and recycling of Adherens Junction, Focal Adhesion, or Extracellular Matrix. **(B)**. Schematic representation of regulatory interaction involving cell mechanics, Autophagy and YAP/TAZ mechano-signaling. Black on white background text indicates the main proteins involved in the regulatory feedbacks.

## 6 Conclusion

This minireview highlights a novel and exciting field of study, the Mechanoautophagy, that aims at understanding how autophagy regulates mechanotransduction machinery and mechanical processes of the cells regulate autophagy. While literature on this topic is in its infancy, this interplay plays an undoubtedly important role during cancer transformation where cancer cells manage to survive in a mechanical microenvironment that in normal conditions would lead to apoptotic clearance. For instance, cancer cells manage to survive in stiff and unstructured ECM, under growing pressure coming from cellular crowding where both cell-substrate and cell-cell adhesions are topologically misconfigured and subject to abnormal forces. Interestingly, escape from programmed cell death is a cancer hallmark that heavily rely on the aid of autophagy. Even more interesting, it has been reported that autophagy provides a mechanism to escape anoikis, i.e., a specific type of apoptosis that lead to clearance of adherence cells lacking proper connection to the ECM. Thus, autophagy is clearly involved in a large number of mechanically related cellular functions, which we only have started to appreciate. Additionally, while we have only discussed the role of mechanoautophagy in cancer transformation, there is a whole plethora of physio/pathological contexts where the study of mechanoautophagy is needed, such as development and/or obesity where the study of autophagy and mechanical forces *per se,* but not their synergy, has been considered. Thanks to technological innovations in creating biomimetic substrates, high temporal and special resolution microscopy as wells the adoption of interdisciplinary approaches, this new field of study could provide fundamental knowledge for a variety of medical conditions. We are thus convinced that better understanding of mechanoautophagy will open the possibility for novel therapeutic interventions targeting for mechanical pathways.
